# Game auction prices are not related to biodiversity contributions of southern African ungulates and large carnivores

**DOI:** 10.1038/srep21922

**Published:** 2016-02-25

**Authors:** Fredrik Dalerum, Maria Miranda

**Affiliations:** 1 Research Unit of Biodiversity (UO-SCIC-PA), University of Oviedo, Campus de Mieres, 33600 Mieres, Spain; 2 Department of Zoology, Stockholm University, 10691 Stockholm, Sweden; 3 Mammal Research Institute, Department of Zoology and Entomology, University of Pretoria, Private Bag X020, 0028 Pretoria, South Africa; 4 Centre for African Ecology, School of Animal, Plant and Environmental Sciences, University of the Witwatersrand, Private Bag 3, Wits 2050, Johannesburg, South Africa; 5 Sports Economics Observatory Foundation (FOED), Department of Economics, University of Oviedo, 33203 Oviedo, Spain

## Abstract

There is an urgent need for human societies to become environmentally sustainable. Because public policy is largely driven by economic processes, quantifications of the relationship between market prices and environmental values can provide important information for developing strategies towards sustainability. Wildlife in southern Africa is often privately owned and traded at game auctions to be utilized for commercial purposes mostly related to tourism. This market offers an interesting opportunity to evaluate how market prices relate to biologically meaningful species characteristics. In this market, prices were not correlated with species contributions to either phylogenetic or functional diversity, and species contributions to phylogenetic or functional diversity did not influence the trends in prices over time for the past 20 years. Since this economic market did not seem to appreciate evolutionary or ecologically relevant characteristics, we question if the game tourism market may contribute towards biodiversity conservation in southern Africa. We suggest that market prices in general may have limited values as guides for directing conservation and environmental management. We further suggest that there is a need to evaluate what humans value in biological organisms, and how potentially necessary shifts in such values can be instigated.

The rapid increase of the human population and its environmental impacts may drive the global environment toward hostile states for our own and other species^1^. Promoting societal change to more sustainable structures is therefore urgent^2^. However, although much of public policy is driven by economic processes, environmental values are still rarely directly included in economic markets^3^. In terms of achieving sustainability goals, this is an obvious shortcoming^4^, and there is subsequently a surging interest in enabling economic valuations of environmental assets^5^. However, the ability of economic markets to contribute to a transition towards sustainability rests on a positive alignment between market forces and long-term environmental goals^6^, which does not always seem to be the case^7^,^8^. Quantifications of the relationship between market forces and environmental values can prove important for our ability to develop strategies to reform human societies towards more sustainable ones^9^.

Throughout southern Africa, wildlife is an important economic asset used in safari tourism and trophy hunting^10^,^11^,^12^, and to a lesser extent also for meat production^13^. However, while these activities may contribute to regional and local economies, they may not always create incentives for ecologically meaningful conservation initiatives^14^. For instance, privately owned game farming may be carried out at ecologically irrelevant scales^15^,^16^. In South Africa, the utilization of wildlife populations on private land is exclusively granted to the land owners^17^. This is an unusual situation since property rights of wildlife are often retained in the public domain^18^. Subsequently, South African wildlife is frequently traded in game auctions for stocking privately owned game reserves^19^. The South African game industry therefore offers an opportunity for evaluating how evolutionary and ecologically relevant characteristics are incorporated in a specific economic market driven by regular market forces. Although meat production is becoming increasingly popular^19^, this market is still driven primarily by revenues from activities related to tourism, including trophy hunting^20^.

Biodiversity is critical for the Earth’s biota, and the recent decline in biodiversity could have dramatic influences for humanity^21^. Although biodiversity was initially measured as species richness, it is now widely accepted that biodiversity consists of several components, ranging from genetic to functional diversity^22^. Genetic diversity reflects the evolutionary history of the organisms within a community, and is often quantified from phylogenetic relationships^23^. Functional diversity, on the other hand, reflects the phenotypic variation of a community that is directly linked to specific ecosystem functions. It therefore reflects contemporary ecosystem performance^24^. Since species differ in both genetic and phenotypic characteristics, there has been a growing awareness of the roles of individual species for the overall diversity of biological communities^25^,^26^.

Here we evaluate the relationship between game auction prices of large herbivore and carnivore species and their contribution to phylogenetic and functional diversity in southern Africa. We calculated two measures of each species’ contribution to its assemblage phylogenetic diversity, evolutionary distinctiveness (ED) and phylogenetic contribution (PC)^26^. Analogously, we quantified contributions to functional diversity as the functional distinctiveness (FD) and functional contribution (FC) of each species. These functional contributions were calculated form dendrograms constructed from matrices of traits related to herbivory (ungulates) and predation (carnivores). We stress that any relationships between prices and these derived metrics are most likely not intentional, i.e. we doubt that market prices are directly related to the importance these species have for different aspects of biodiversity. However, a positive relationship would suggest that the characteristics that are valued by this market are indeed positively correlated with evolutionary and ecologically relevant characteristics. Such correlations may be necessary for this market to positively contribute to biodiversity conservation and sustenance in southern Africa. We restricted our analyses to the southern African ungulate and large carnivore assemblages, and only included species naturally occurring within the southern African subregion^27^. The ungulate assemblage consisted of all terrestrial species within the region belonging to the mammalian order Perissodactyla and the superorder Cetartiodactyla, while the carnivore assemblage contained species over 10 kg in size within the order Carnivora^26^. These definitions yielded total assemblages of 41 ungulate and 13 carnivore species, of which we had prices for 37 and 6 species, respectively.

## Results

**Figure 1:**
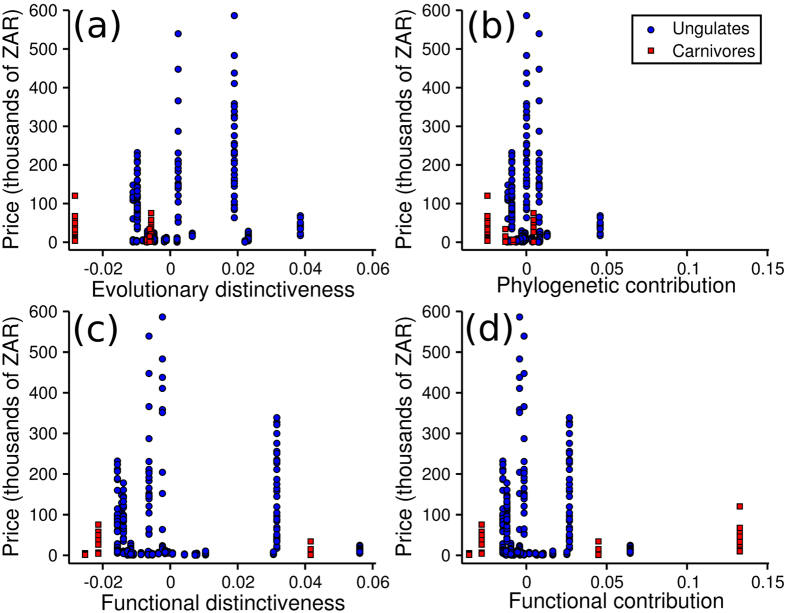
Relationships between game auction prices of southern African ungulates and carnivores and their evolutionary distinctiveness (**a**), phylogenetic contribution (**b**), functional distinctiveness (**c**), and functional contribution (**d**). Prices were adjusted by the change in the consumer price index using 2012 as comparison year. Phylogenetic and functional metrics have been scaled to represent the deviation from equal contributions of each species.

Game auction prices showed negative but non-significant relationships with contributions to phylogenetic diversity (ED β = −28.98, t_1_ = 0.43, p = 0.667; PC β = −7.07, t_1_ = 0.10, p = 0.920; Fig. 1a,b), and non-significant positive relationships with functional diversity (FD β = 1.43, t_1_ = 0.10; p = 0.922; FC β = 2.64, t_1_ = 0.19, p = 0.847; Fig. 1c,d). The effects of diversity contributions on prices did not differ significantly between the two taxonomic groups, either for phylogenetic (ED β = 60.26, t_1_ = 0.85, p = 0.398; PC, β = 23.66, t_1_ = 0.32, p = 0.752) or functional (FD β = −12.34, t_1_ = 0.54, p = 0.594; FC β = −5.56, t_1_ = 0.25, p = 0.804) contributions.

**Figure 2:**
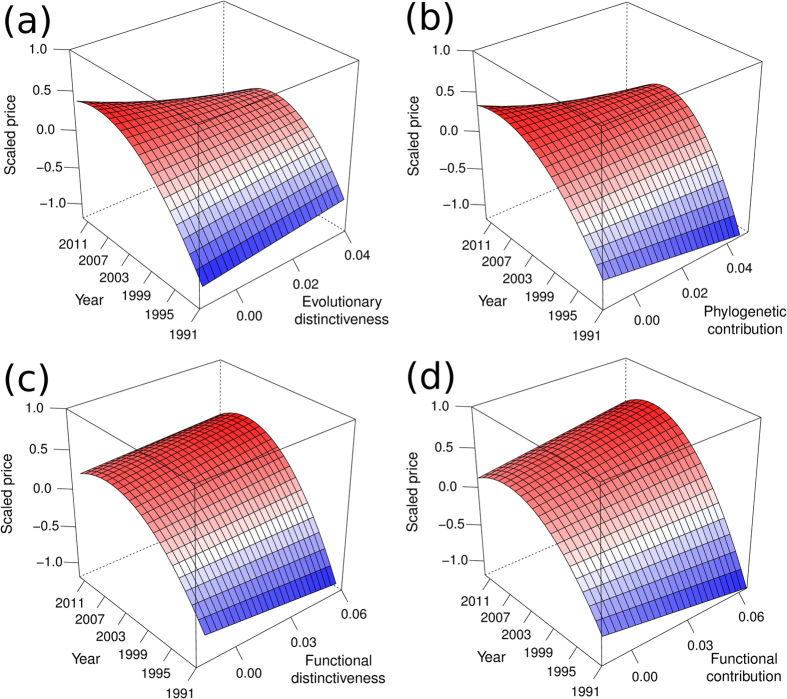
Relationships between predicted scaled prices for southern African ungulates from 1991 to 2012 and their evolutionary distinctiveness (**a**), phylogenetic contribution (**d**), functional distinctiveness (**c**), and functional contribution (**d**). Prices are shown as the deviation from the mean price of each species, and are in units of standard deviations from this mean. The relationships are quantified as coefficients from linear mixed models using a quadratic relationship between scaled price and time. Prices were adjusted by the change in the consumer price index using 2012 as comparison year prior to being adjusted for species specific means and scaled by species specific standard deviations.

Scaled prices of ungulates showed a humped trend over time (time β = 0.15, t_1_ = 5.36, p < 0.001, time^2^ β = −0.01, t_1_ = 3.77, p < 0.001), with an increase in scaled prices until after the millennium, and a subsequent decline in the latter half of the 2000’s (Fig. 2a–d). Neither phylogenetic (ED χ^2^_2_ = 1.96, p = 0.376; PC χ^2^_2_ = 0.69, p = 0.708 ; Fig. 2a,b) nor functional (FD χ^2^_2_ = 0.26, p = 0.878; FC χ^2^_2_ = 0.65, p = 0.724 ; Fig. 2c,d) diversity contributions significantly influenced this relationship.

## Discussion

Our study failed to find any significant relationships between game auction prices and the evolutionary or ecological significance of southern African ungulates and large carnivores. Hence, our results indicate that these market prices were driven by attributes, most likely aesthetic and cultural^28^, which are not directly linked to either evolutionary history or ecologically relevant characteristics. Considering that these animals mostly are traded for inclusion in profit driven game reserves, we suggest that the prices in this market at least partially reflect the relative public demand for the different species^11^. Such an interpretation would suggest a lack of appreciation of the relative evolutionary or ecological importance among these species, which could lead to serious mis-allocations of resources towards the management and protection of biological resources^29^. However, we point out that this market only reflects a section of society that is interested in game related tourism and also has the financial assets to pay for it. Although this is an obvious limitation of the data, we argue that the results still may have significant ramifications for biodiversity conservation in southern Africa.

Despite an increased attention to environmental degradation and the importance of Earth’s biota^2^, species importance for either phylogenetic or functional diversity did not influence the temporal trends in game auction prices from 1991 to 2012. Hence, the expansion in public attention to environmental problems does not appear to have influenced the relative market prices of these species. This is an interesting observation, since market prices partially are influenced by consumer preferences. The observed lack of effect of phylogenetic or ecological characteristics on the temporal trend in prices may therefore support previous studies pointing to a discrepancy between peoples’ recognition of environmental problems and their inclination to act according to that knowledge^30^,^31^.

Our two assemblages included some of the most recognized flagship species in the world^15^,^32^. In addition, our study only included species in a region where conservation action is claimed to successfully have merged with market interests, and where a major proportion of conservation activities occurs outside of formally protected areas^20^,^33^. Or results therefore support some previous concerns that market interests may not be useful as foundations for evolutionary and ecologically meaningful preservation and management of biological resources^34^,^35^,^36^,^37^. We appreciate that the failure of this particular market to recognize the evolutionary and ecological significance of African ungulates and carnivores could partly have been caused by difficulties to directly perceive it. However, if the species attributes that were appreciated in the market were not correlated with evolutionary or ecologically relevant characteristics, it is questionable if such attributes should direct conservation and environmental management.

Since the values in economic markets to some extent reflect human preferences^38^, we suggest that our results may imply a need for altering tourism preferences for wildlife species to better align with their biological relevance. This may be necessary as a step towards enabling a broad social acceptance of implementations of policies for environmental management that target biologically important organisms and processes^6^,^39^. However, an increased public appreciation of the biological significance of individual species could lead to a situation where important species suffer elevated extinction risks due to an increased desirability for them^40^,^41^,^42^,^43^. Therefore, any efforts to change public opinion must be accompanied by appropriate changes in regulatory and educational structures, to ensure that shift in values permeate all components of society. A lack of awareness could also lead to a discrepancy between the demand for conservation aligned alternatives and their availability, if any shifts in public attitudes are not fully perceived by the producers^11^.

## Methods

### Data on market prices of species

We compiled the average prices for each year between 1991 and 2012 from information in the recreational journal *Game & Hunt* (http://www.wildlifehunt.co.za) and *African Indaba*, an electronic newsletter from International Council for Game and Wildlife Conservation (http://africanindaba.com). Unfortunately only the average price for each species (or in some cases subspecies or deviating colour morphs) were available from this source, and not the original prices or the number of sales the averages had been calculated from. In total there were prices available for 37 species in the ungulate assemblage and 6 species in the carnivore assemblage (Supplementary Table S1). We excluded the subspecies Cape mountain zebra (*Equus zebra zebra*), Livingstone’s eland (*Taurotragus oryx livingstonii*) and bontebok (*Damaliscus pygargus pygarus*) as well as animals with deviating colour morphs, as we did not regard them to be representative for the species. In cases were there were different prices for males and females we averaged the price across both sexes. To allow comparisons across time we adjusted the prices for inflation using consumer price indices (CPI) for South Africa (available at http://global-rates.com, the available indices are based on figures from the South African Reserve Bank). We calculated annual inflation as the changes between years in the December estimates of CPI, and used prices adjusted to 2012 year’s level in subsequent analyses (Supplementary Table S2).

### Quantifications of species contributions to phylogenetic diversity

For quantifications of species contributions to phylogenetic diversity, we used a near complete species-level phylogeny for Mammalia^43^. We created two cropped trees, one for each of the two assemblages (Supplementary Figure S1). We let these trees represent the overall phylogenetic diversity of southern African ungulates and large carnivores.

ED was calculated as the sum of all branches along each species evolutionary trajectory after weighting each branch by the number of species sharing it^44^, and PC as the length of a species’ terminal branch (Supplementary Tables S7and S8). Both ED and PC were scaled by the total phylogenetic diversity contained within each assemblage, quantified as the sum of all branch lengths in the respective phylogenetic tree. To enable direct comparisons between the ungulate and carnivore assemblages, which contained different number of species, we expressed species contributions as the deviation from equal contributions of all species^26^.

### Quantifications of species contributions to functional diversity

We focused on quantifying contributions to functional diversity with regard to herbivory (ungulate assemblage) and predation (carnivore assemblage) processes. We quantified the functional diversity of each assemblage from dendrograms constructed from matrices of relevant functional traits^45^.

For the ungulate assemblage, we compiled a trait matrix containing adult body mass, feeding type, diet breadth, habitat breadth, population density and social group size (Supplementary Tables S3 and S4). We used traits available in the PanTHERIA 1.0 WR05 database on mammal traits^46^, which follows the taxonomy by Wilson and Reeder^47^. Missing data where extracted from Skinner and Smithers^27^, the IUCN Red List (http://www.iucnredlist.org/), Mammalian Species, Leuthold^48^ and the Antelope and Giraffe Taxon Advisory Group (Supplementary Table S3). The trait matrix for the carnivore assemblage included diet, body size, hunting group size, area use and a palaeo-morphological classification of carnivore functional groups (Supplementary Tables S5 and S6). Traits and data sources are described in detail elsewhere^26^.

To create the dendrograms, we first standardized numeric variables so that they had a mean of 0 and a standard deviation of 1. We then created distance matrices from each trait matrix using Gower’s distance method^49^, and clustered these distance matrices into one dendrogram for each assemblage using the UPGMA (Unweighted Pair-Group Method using Arithmetic averages) clustering method (Supplementary Figure S2). We choose this method since it yielded the highest cophenetic correlation^50^.

Analogous to how we quantified contributions to phylogenetic diversity, we determined FD for each species as the sum of all contributed branches where each branch was weighted by the number of species sharing it, and FC as the length of each species terminal branch in the functional tree (Supplementary Tables S7 and S8)^26^. We scaled FD and FC by the total trait variation in each guild, quantified as the sum of all branch lengths in each dendrogram, so that species contributions were proportional to the total functional diversity contained within each guild. As with phylogenetic contributions, we expressed these proportional contributions as deviations from equal contributions of all species.

### Data analyses

We used linear mixed models to relate phylogenetic and functional diversity contributions of species to their recorded auction prices. Since ED and PC as well as FD and FC were highly correlated (ED vs PC, R^2^ = 0.77; FD vs FC, R^2^ = 0.97), we created two separate models, one for overall diversity contributions using only ED and FD, and one for unique contributions using PC and FC. In both models, we used the annual price recorded for each species from 1991 and 2012 as response variable, taxonomic group (i.e. ungulates and carnivores) as categorical predictor and phylogenetic and functional contributions as continuous predictors. We also added the two-way interaction effects between taxonomic group and phylogenetic and functional contributions as an evaluation of differences in biodiversity contribution-price relationships between ungulates and carnivores. Since these models included longitudinal data on each species, we added year of recorded price grouped across species as a random effect structure.

We similarly explored the effect of phylogenetic and functional contributions on the temporal trends in prices using linear mixed models. For these models, we calculated the deviation from the mean price for the whole time period for a given species and year, and normalized these deviations for all species by dividing by each species standard deviation. This scaled variable hence represents the deviation in units of standard deviations from the average price for a given year. We used this scaled variable as response variable in the analyses. Because we only had data for a limited number of years for most carnivore species, we restricted this analysis to ungulates. Similarly to the previous analyses, we fitted two models, one including overall (ED and FD) and one including unique (PC and FC) contributions as predictors. Visual inspections suggested a non-linear relationship between time and normalized price deviations. We therefore tested linear, quadratic and cubic relationship between time and price. Of these, a quadratic relationship provided the best fit (cubic vs. quadratic χ^2^_2_ = 2.59, p = 0.107; quadratic vs. linear χ^2^_2_ = 13.05, p = 0.004). Therefore, for each model we also included time as a fixed co-variate modelled as a second order polynomial, and we fitted two-way interactions of the two diversity contributions and both of the polynomial time parameters. For each model we added species identity as a random term and accounted for temporal correlation by adding a first order autocorrelation structure^51^.

All statistical analyses were conducted using the statistical software R version 3.2.2 for Linux (http://www.r-project.org). Phylogenetic calculations were conducted using the Tuatara package version 1.01 for the phylogenetic software Mesquite (version 2.75)^52^,^53^. We used the contributed packages picante^54^ and cluster^55^ to calculate functional diversity metrics, and the functions in package lme^56^ to conduct the linear modelling.

## Additional Information

**How to cite this article**: Dalerum, F. and Miranda, M. Game auction prices are not related to biodiversity contributions of southern African ungulates and large carnivores. *Sci. Rep.* **6**, 21922; doi: 10.1038/srep21922 (2016).

## Supplementary Material

Supplementary Information
