# Supplementary material for: Game auction prices are not related to biodiversity contributions of southern African ungulates and large carnivores
Source: Sci Rep. 2016 Feb 25;6:21922. doi: 10.1038/srep21922 (PMC4766510; doi:10.1038/srep21922)
Supplement: Supplementary Information [file srep21922-s1.pdf]

Game auction prices are not related to biodiversity contributions of southern African ungulates and large carnivores

Fredrik Dalerum and Maria Miranda

Supplementary Tables and Figures, *Scientific Reports* 6:21922

Table S1. Raw annual auction prices (ZAR) for southern African mammals from 1991 to 2012.

Species	1991[1]	1992[1]	1993[1]	1994[1]	1995[1]	1996[1]	1997[1]	1998[1]	1999[1]	2000[1]	2001[2]	2002[2]	2003[3]	2004[3]	2005[3]	2006[4]	2007[5]	2008[6]	2009[7]	2010[8]	2011[9]	2012[10]
UNGULATES																						
Aepyceros melampus	200	211	233	325	369	480	627	579	567	596	642	724	675	638	725	754	982	1153	1099	1122	1160	1122
Alcelaphus buselaphus	1150	907	1006	1016	1678	1956	2485	2751	2722	2960	3189	4331	3483	3336	3122	3311	3861	4138	4409	3971	4314	3828
Alcelaphus lichtensteinii						25750		30000					75000	70000			95714	90000	100000		140000	
Antidorcas marsupialis	118	168	268	243	303	305	403	473	410	382	501	652	603	523	603	779	616	1108	866	980	1392	1451
Cephalophus natalensis	2125	1725	1450					175	2000		2600		4800		4500		3000					
Ceratotherium simum	43800	29375	28057	32306	46629	44575	74309	112153	126583	176801	169300	206060	148133	112750	95281	128897	204443	274712	220064	223099	199794	231807
Connochaetes gnou	447	465	599	1049	1404	1646	2219	2596	2551	2681	2313	2684	1639	1797	1471	2112	3019	2825	2435	2154	2970	2192
Connochaetes taurinus	744	490	816	1441	1393	1783	2226	2129	2248	2226	2326	2269	1785	1631	1634	1783	2276	2617	2439	2374	2907	2156
Damaliscus lunatus		3057	2982	2740	2453	3423	4706	5906	6365	9500	15285	17111	16739	19123	15321	10182	12693	12320	11280	12127	11832	14317
Damaliscus pygargus (blesbok)	270	255	290	382	435	565	613	672	651	676	703	829	743	747	723	942	1161	1156	1328	1262	1122	1226
Diceros bicornis	245000	460000	195000	150000	140000	150000			221000	375000	550000	451660	40000	131667	100000	333125						
Equus quagga	1452	1052	1150	1416	1491	1716	2088	2321	2219	2640	3006	4545	4543	4590	4736	5025	4894	5265	5248	4550	4826	4262
Equus zebra						2740	2388	2580	3000		9000	14467	18000	17953	11595	13125	11233	10596	16688	12250	10127	9108
Giraffa camelopardalis	6584	6050	6040	6284	7005	7205	9323	10263	10993	12509	13118	14776	14506	13993	13696	15498	13673	16118	13647	18264	14187	15678
Hippopotamus amphibius		5200			15000			18247	23500	25300	27000	44111	40000	31625	28174	15500	25034	41000		43144	48125	34500
Hippotragus equinus	17000	29000		28000	38500	33625	26400	48500	94097	86389	106714	135650	130333	149521	66417	60903	67532	67738	62993	67178	108219	223650
Hippotragus niger	25286	18281	19163	15667	13480	17250	24892	32460	48774	53580	66534	78360	91561	61814	47224	57415	63607	71462	121827	117731	152122	178121
Kobus ellipsiprymnus	2100	1785	1544	1902	2185	2607	3358	3564	3537	4828	5060	6656	5563	4989	4786	5398	6095	6197	6006	6269	5130	4311
Kobus leche		4250	2300	1800	1972	2617	3500	4434	3928	5752	7275	11535	15491	14009	10978	9730	11350	9946	10175	10369	8625	8533
Neotragus moschatus		1333	2050	1300			2000						3957					5511				
Oreotragus oreotragus		2300	2400	2459			4363	5050	5986	5600	4918	5555	4725	3833	4900	3150					10000	10000
Oryx Gazella	1248	1044	1136	1414	1881	1981	2635	2946	3146	3295	3388	3745	3762	3519	3443	4091	4011	4310	4762	4757	4934	4860
Ourebia ourebi		3283	2600	1786	2450		2500	2500	2723		3800	7500		5000								
Pelea capreolus	205	688	735			750	3900	2177	1900		2035	3200	4000	4333	4316		7250			2000		
Phacochoerus africanus		267	650				500	857	621	567	719	896	718	720	646	661	790	533	1133	710	1306	1000
Philantomba monticola	1700	520	840			2000	400	1611					2800	3700							10000	
Potamochoerus larvatus					100	1250			800		800	300	440	2700	300						400	600
Raphicerus campestris	312	300	172	1180	586	1368	1107	1048	2175	1009	1510	1850	1659	1305	1575	1666	1450	2100	1761	5012	4438	4355
Raphicerus melanotis		235							1657		2000	500	1700		3100	1650						
Redunca arundinum	1450	574	602	1500	1400	1959	2315	2552	2272	3400	3768	3802	4459	4419	3348	3462	4694	5515	4218	4650	5000	7299
Redunca fulvorufula	249	476	334	483	621	642	1301	1478	813	993	1247	1252	1460	1274	1063	1531	1949	2159	1910	3097	2857	3110
Sylvicapra grimmia	400	563	370	515	588	1500	724	872	870	1149	1302	1165	1049	2191		1503	1459		1183	811	2790	1941
Syncerus caffer	28812	16391	17250	23750	47163	65038	74854	101784	74607	114246	81409	117491	120890	148867		95926	108253	164126	246878	325282	510225	447494
Taurotragus oryx	2293	1735	1978	1997	2737	3016	4025	4337	4160	4719	4524	5146	4475	4390	4476	5363	5633	6605	6879	7153	6711	5473
Tragelaphus angasii	1909	1483	1323	2095	2176	2619	3129	3708	3285	5924	7123	5490	6617	6503	5462	5480	6635	7217	7455	6979	6809	7686
Tragelaphus scriptus	1500	669	645	1544	1481	1244	2060	2742	2078	2575	2767	4737	2725	2429	2111	3325	3276	4073	4057	3278.5	3853	8470.5
Tragelaphus strepsiceros	868	822	878	1205	1347	1854	1889	1651	1813	2105	2323	2294	2211	2032	2294	2641	4009	4991	4894	5417	5089	4124
CARNIVORES																						
Acinonyx jubatus														32000		26250		47071	50000	67292	25000	
Caracal caracal															3500			1600	2375			1200
Crocuta crocuta														500				5600	5600			
Lycaon pictus						3000	15500	4250			7325	8000	9500					2000	1013			
Panthera leo		8670	7000	7833	4083	14750		25429	18166	8200	15000	36400	22991	6314	21833		7429	97500	28469		64025	46000
Panthera pardus			2250								2250				32667							

Sources: 1) *Game & Hunt* Dec 2000; 2) *African Indaba* Mar 2003; 3) *Game & Hunt* Feb 2006; 4) *Game & Hunt* March 2007; 5) *Game & Hunt* Feb 2008; 6) *Game & Hunt* Feb 2009; 7) *Game & Hunt* Feb 2010; 8) *Game & Hunt* Feb 2011; 9) *Game & Hunt* Feb 2012; 10) *Game & Hunt* Mar 2013

Table S2. Annual auction prices adjusted for inflation (ZAR) using 2012 as reference year. Inflation corrections were based on consumer price indices (CPI) for South Africa available from http://global-rates.com and are based on figures from the South African Reserve Bank. Annual inflation was calculated as the annual changes between December estimates of CPI.

Species	1991	1992	1993	1994	1995	1996	1997	1998	1999	2000	2001	2002	2003	2004	2005	2006	2007	2008	2009	2010	2011	2012
UNGULATES																						
Aepyceros melampus	721	695	701	890	945	1124	1383	1172	1123	1103	1136	1129	1070	990	1102	1094	1324	1422	1278	1262	1226	1122
Alcelaphus buselaphus	4148	2988	3025	2782	4296	4581	5482	5571	5391	5479	5644	6753	5521	5174	4746	4802	5206	5102	5127	4467	4560	3828
Alcelaphus lichtensteinii						60313		60747					118882	108568			129050	110970	116277		147994	
Antidorcas marsupialis	426	553	806	665	776	714	889	958	812	707	887	1017	956	811	917	1130	831	1366	1007	1102	1471	1451
Cephalophus natalensis	7665	5682	4360					354	3961		4602		7608		6841		4045					
Ceratotherium simum	157999	96762	84364	88446	119385	104406	163935	227099	250702	327284	299645	321299	234804	174871	144852	186946	275647	338720	255883	250955	211202	231807
Connochaetes gnou	1612	1532	1801	2872	3595	3855	4895	5257	5052	4963	4094	4185	2598	2787	2236	3063	4070	3483	2831	2423	3140	2192
Connochaetes taurinus	2684	1614	2454	3945	3567	4176	4911	4311	4452	4121	4117	3538	2829	2530	2484	2586	3069	3227	2836	2670	3073	2156
Damaliscus lunatus		10070	8966	7501	6280	8018	10382	11959	12606	17586	27053	26680	26533	29659	23292	14767	17114	15191	13116	13641	12508	14317
Damaliscus pygargus (blesbok)	974	840	872	1046	1114	1323	1352	1361	1289	1251	1244	1293	1178	1159	1099	1366	1565	1425	1544	1420	1186	1226
Diceros bicornis	883783	1515248	586338	410661	358444	351337			437699	694179	973448	704251	63403	204211	152026	483148						
Equus quagga	5238	3465	3458	3877	3817	4019	4606	4700	4395	4887	5320	7087	7201	7119	7200	7288	6599	6492	6102	5118	5102	4262
Equus zebra						6418	5268	5224	5942		15929	22558	28532	27844	17627	19036	15145	13065	19404	13780	10705	9108
Giraffa camelopardalis	23750	19929	18161	17204	17935	16876	20568	20782	21772	23156	23218	23039	22993	21703	20821	22478	18435	19873	15868	20544	14997	15678
Hippopotamus amphibius		17129			38405			36948	46543	46834	47787	68780	63403	49049	42832	22480	33753	50553		48531	50873	34500
Hippotragus equinus	61324	95527		76657	98572	78758	58242	98208	186363	159918	188874	211512	206589	231902	100971	88331	91052	83521	73246	75566	114398	223650
Hippotragus niger	91214	60218	57621	42892	34513	40404	54915	65728	96599	99184	117759	122183	145132	95871	71793	83272	85760	88113	141657	132431	160808	178121
Kobus ellipsiprymnus	7575	5880	4643	5207	5594	6106	7408	7217	7005	8937	8956	10378	8818	7738	7276	7829	8218	7641	6984	7052	5423	4311
Kobus leche		14000	6916	4928	5049	6130	7721	8978	7780	10648	12876	17986	24555	21727	16689	14112	15303	12263	11831	11664	9117	8533
Neotragus moschatus		4391	6164	3559			4412						6272					6795				
Oreotragus oreotragus		7576	7216	6732			9625	10226	11856	10366	8704	8662	7490	5945	7449	4569					10571	10000
Oryx Gazella	4502	3439	3416	3871	4816	4640	5813	5965	6231	6100	5996	5839	5963	5458	5234	5933	5408	5314	5537	5351	5216	4860
Ourebia ourebi		10814	7818	4890	6273		5515	5062	5393		6726	11694		7755								
Pelea capreolus	739	2266	2210			1757	8604	4408	3763		3602	4990	6340	6720	6561		9775			2250		
Phacochoerus africanus		880	1954				1103	1735	1230	1050	1273	1397	1138	1117	982	959	1065	657	1317	799	1381	1000
Philantomba monticola	6132	1713	2526			4684	882	3262					4438	5739							10571	
Potamochoerus larvatus					256	2928			1584		1416	468	697	4188	456						423	600
Raphicerus campestris	1125	988	517	3231	1500	3204	2442	2122	4308	1868	2673	2885	2630	2024	2394	2416	1955	2589	2048	5638	4691	4355
Raphicerus melanotis		774							3282		3540	780	2695		4713	2393						
Redunca arundinum	5231	1891	1810	4107	3584	4588	5107	5168	4500	6294	6669	5928	7068	6854	5090	5021	6329	6800	4905	5231	5286	7299
Redunca fulvorufula	898	1568	1004	1322	1590	1504	2870	2993	1610	1838	2207	1952	2314	1976	1616	2220	2628	2662	2221	3484	3020	3110
Sylvicapra grimmia	1443	1855	1113	1410	1505	3513	1597	1766	1723	2127	2304	1817	1663	3398		2180	1967		1376	912	2949	1941
Syncerus caffer	103933	53992	51868	65021	120752	152335	165138	206103	147762	211486	144086	183198	191621	230888		139126	145956	202367	287062	365897	539359	447494
Taurotragus oryx	8271	5715	5948	5467	7008	7064	8880	8782	8239	8736	8007	8024	7093	6809	6805	7778	7595	8144	7999	8046	7094	5473
Tragelaphus angasii	6886	4885	3978	5736	5571	6134	6903	7508	6506	10966	12607	8560	10489	10086	8304	7948	8946	8899	8668	7850	7198	7686
Tragelaphus scriptus	5411	2204	1939	4227	3792	2914	4545	5552	4116	4767	4897	7386	4319	3767	3209	4822	4417	5022	4717	3688	4073	8471
Tragelaphus strepsiceros	3131	2708	2640	3299	3449	4343	4167	3343	3591	3897	4111	3577	3505	3152	3487	3830	5405	6154	5691	6093	5380	4124
CARNIVORES																						
Acinonyx jubatus														49631		38072		58038	58138	75694	26428	
Caracal caracal															5321			1973	2762			1200
Crocuta crocuta														775				6905	6512			
Lycaon pictus						7027	34195	8606			12965	12474	15058					2466	1178			
Panthera leo		28559	21048	21445	10454	34548		51491	35978	15179	26549	56757	36443	9793	33192		10016	120217	33103		67681	46000
Panthera pardus			6765								3982				49662							

Table S3. Traits used to quantify functional diversity in the ungulate assemblage.

Trait	Explanation	Data sources
Body mass	Average adult body mass (kg)	Jones et al. 2009
Feeding type	Categorical variable describing mode of herbivory:<br>Grazer<br>Browser<br>Mixed feeder	Skinner and Smithers 1990
Diet breadth	Numeric value describing the number of dietary categories eaten by each species. Categories were defined as vertebrate, invertebrate, fruit, flowers/nectar/pollen, leaves/branches/bark, seeds, grass and roots/tubers.	Skinner and Smithers 1990
Habitat breadth	Numeric value describing the number of habitat layers used by each specie. Categories were defined as above ground dwelling, aquatic, fossorial and ground dwelling.	Jones et al. 2009 except<br>*Damaliscus pygargus* Skinner and Smithers 1990<br>*Phacochoerus africanus* Skinner and Smithers 1990
Density	Average density (animals/100 km^2)	Jones et al. 2009 except<br>*Phacochoerus africanus* Skinner and Smithers 1990<br>*Tragelaphus angasii* Richard and Shurter 2006
Group size	Average social group size	Jones et al. 2009 except<br>*Antidorcas marsupialis* Cain et al. 2004<br>*Damaliscus pygargus* Skinner and Smithers 1990<br>*Phacochoerus africanus* Skinner and Smithers 1990<br>*Giraffa camelopardalis* Leuthold 1979<br>*Hippopotamus amphibius* Skinner and Smithers 1990<br>*Equus quagga* Skinner and Smithers 1990

Table S4. Functional trait matrix for the ungulate assemblage.

Species	Body mass	Feeding type	Diet breath	Habitat breadth	Density	Group size
Aepyceros melampus	52	mixed	3	1	13.36	15.25
Alcelaphus buselaphus	161	grazer	2	1	3.65	20.00
Alcelaphus lichtensteinii	169	grazer	2	1	1.71	10.00
Antidorcas marsupialis	34	mixed	4	1	10.69	34.00
Cephalophus natalensis	13	browser	4	1	6.92	2.00
Ceratotherium simum	2286	grazer	2	1	0.47	1.00
Connochaetes gnou	157	grazer	2	1	34.62	26.75
Connochaetes taurinus	199	grazer	1	1	7.99	20.00
Damaliscus lunatus	136	grazer	1	1	2.74	3.60
Damaliscus pygargus	78	grazer	1	1	24.56	11.00
Diceros bicornis	996	browser	2	1	0.11	1.00
Equus quagga/burchelii	279	grazer	4	1	7.00	5.00
Equus zebra	282	grazer	2	1	1.32	13.00
Giraffa camelopardalis	965	browser	4	1	1.23	3.80
Hippopotamus amphibius	1536	grazer	2	2	4.86	3.00
Hippotragus equinus	264	grazer	2	1	0.96	12.00
Hippotragus niger	236	grazer	3	1	1.30	15.00
Kobus ellipsiprymnus	204	grazer	3	1	2.46	12.00
Kobus leche	89	grazer	2	2	31.00	20.00
Kobus vardonii	71	grazer	2	1	4.78	5.50
Madoqua kirkii	5	browser	4	1	19.00	2.50
Neotragus moschatus	6	browser	4	1	17.32	1.50
Oreotragus oreotragus	13	browser	4	1	2.62	2.00
Oryx gazella	188	grazer	4	1	9.88	20.00
Ourebia ourebi	17	mixed	2	1	2.43	2.00
Pelea capreolus	23	browser	5	1	18.71	10.25
Phacochoerus africanus	82	omnivore	7	1	5.00	3.00
Philantomba monticola	5	browser	3	1	35.98	1.90
Potamochoerus larvatus	69	omnivore	7	1	0.40	8.00
Raphicerus campestris	12	browser	5	1	1.27	2.00
Raphicerus melanotis	11	browser	2	1	10.50	1.50
Raphicerus sharpei	9	browser	3	1	0.51	1.50
Redunca arundinum	58	grazer	2	1	0.65	1.75
Redunca fulvorufula	29	grazer	1	1	7.77	4.75
Sylvicapra grimmia	16	browser	8	1	2.42	1.00
Syncerus caffer	593	grazer	1	1	5.84	12.00
Taurotragus oryx	563	mixed	3	1	1.64	4.05
Tragelaphus angasii	88	mixed	4	1	1.00	2.50
Tragelaphus scriptus	43	browser	4	2	1.52	1.00
Tragelaphus spekii	76	mixed	2	2	1.66	6.00
Tragelaphus strepsiceros	206	browser	3	1	1.06	12.00

Table S5. Traits used to quantify functional diversity in the large carnivore assemblage. Data sources are given in Dalerum 2013

Trait	Explanation
Functional group[a]	Categorical classification of functional groups:<br>Scavenger/Omnivore<br>Bone-crushing<br>Bone-cracking<br>Stalk and ambush<br>Pursuit
Home range size	Average home range size recorded for the species (km^2)
Density	Average density recorded for the species (animals/100 km^2)
Diet	Categorical classification of main diet:<br>Carnivore<br>Insectivore<br>Piscivore<br>Herbivore<br>Omnivore
Mean prey size	Average recorded prey size for the species (kg)
Max prey size	Maximum recorded prey size for the species (kg)
Min prey size	Minimum recorded prey size for the species (kg)
Group size	Average recorded hunting group size for the species
Adult body mass	Average adult body mass recorded for the species (kg)
Sexual dimorphism Male:Female	Sexual dimorphism in body weight recorded for the species (ratio of male to female)

a) Functional groups defined from Werdelin 1996

Table S6. Functional trait matrix for the carnivore assemblage.

Species	Functional group	Home range size	Density	Diet	Max prey size	Min prey size	Mean prey size	Group size	Body mass	Sex dimorph (male:female)
Acinonyx jubatus	SA	300	0.5	carnivore	200	1	40	1	40	1.1
Canis adustus	P	10	100	omnivore	5	0.01	0.1	1	9	1.1
Canis mesomelas	P	8	40	omnivore	40	0.01	1	2	8.5	1.1
Caracal caracal	SA	10	10	carnivore	30	0.1	0.5	1	11.5	1.3
Civettictis civetta	SO	10	10	omnivore	1	0.001	0.1	1	12	1
Crocuta crocuta	BCa	500	35	carnivore	500	0.1	40	15	55	0.85
Leptailurus serval	SA	12.5	40	carnivore	1	0.1	0.1	1	11	1.2
Lycaon pictus	BCu	600	2	carnivore	250	3	50	10	25	1
Mellivora capensis	SO	300	10	omnivore	1	0.01	0.1	2	9	1.3
Panthera leo	SA	1000	10	carnivore	500	5	150	3	150	1.5
Panthera pardus	SA	500	2	carnivore	200	1	20	1	60	1.3
Parahyaena brunnea	BCa	300	2	omnivore	10	0.1	0.5	1	40	1
Proteles cristatus	SO	3	50	insectivore	0.01	0.001	0.001	1	10	1

Table S7. Total branch length weighted for number of species contributing to each segment (ED and FD) and terminal branch lengths (PC and FC) calculated from a phylogenetic tree and a functional dendrogram for 41 species of southern African ungulates. The branch length values were used to calculate species contributions to evolutionary distinctiveness (ED), phylogenetic contribution (PC), functional distinctiveness (FD) and functional contribution (FC) in the southern African ungulate assemblage.

Species	ED	PC	FD	FC
Aepyceros melampus	27.030	23.000	0.090	0.070
Alcelaphus buselaphus	13.670	2.800	0.050	0.020
Alcelaphus lichtensteinii	13.670	2.800	0.030	0.010
Antidorcas marsupialis	27.030	23.000	0.090	0.070
Cephalophus natalensis	17.880	8.100	0.030	0.010
Ceratotherium simum	44.410	14.700	0.160	0.130
Connochaetes gnou	13.970	3.400	0.100	0.080
Connochaetes taurinus	13.970	3.400	0.050	0.020
Damaliscus lunatus	18.070	11.600	0.040	0.020
Damaliscus pygargus	18.070	11.600	0.100	0.080
Diceros bicornis	44.410	14.700	0.070	0.030
Equus quagga/burchelii	48.660	23.200	0.060	0.050
Equus zebra	48.660	23.200	0.030	0.000
Giraffa camelopardalis	31.660	27.800	0.070	0.030
Hippopotamus amphibius	64.460	61.600	0.160	0.130
Hippotragus equinus	14.980	5.300	0.030	0.000
Hippotragus niger	14.980	5.300	0.030	0.010
Kobus ellipsiprymnus	20.040	13.800	0.030	0.010
Kobus leche	20.040	13.800	0.240	0.240
Kobus vardonii	20.190	14.100	0.040	0.020
Madoqua kirkii	27.030	23.000	0.040	0.010
Neotragus moschatus	27.030	23.000	0.040	0.010
Oreotragus oreotragus	27.030	23.000	0.030	0.010
Oryx gazella	16.630	8.600	0.050	0.040
Ourebia ourebi	27.030	23.000	0.070	0.030
Pelea capreolus	23.200	18.400	0.050	0.040
Phacochoerus africanus	47.640	22.100	0.090	0.020
Philantomba monticola	20.230	12.800	0.100	0.100
Potamochoerus larvatus	47.640	22.100	0.090	0.020
Raphicerus campestris	13.670	2.400	0.040	0.020
Raphicerus melanotis	14.770	4.600	0.060	0.040
Raphicerus sharpei	13.670	2.400	0.040	0.020
Redunca arundinum	17.720	8.100	0.040	0.020
Redunca fulvorufula	17.720	8.100	0.040	0.020
Sylvicapra grimmia	17.880	8.100	0.080	0.080
Syncerus caffer	27.370	22.700	0.050	0.040
Taurotragus oryx	18.970	8.600	0.070	0.040
Tragelaphus angasii	23.770	18.200	0.070	0.030
Tragelaphus scriptus	20.020	10.700	0.160	0.120
Tragelaphus spekii	20.020	10.700	0.160	0.120
Tragelaphus strepsiceros	18.970	8.600	0.070	0.060

Table S8. Total branch length weighted for number of species contributing to each segment (ED and FD) and terminal branch lengths (PC and FC) calculated from a phylogenetic tree and a functional dendrogram for 13 species of southern African large carnivores. The branch length values were used to calculate species contributions to evolutionary distinctiveness (ED), phylogenetic contribution (PC), functional distinctiveness (FD) and functional contribution (FC) in the southern African large carnivore assemblage.

Species	ED	PC	FD	FC
Acinonyx jubatus	22.085	0.071	0.197	0.080
Canis adustus	19.400	0.063	0.196	0.076
Canis mesomelas	19.400	0.063	0.196	0.076
Caracal caracal	22.085	0.071	0.184	0.052
Civettictis civetta	39.400	0.127	0.193	0.085
Crocuta crocuta	21.708	0.070	0.421	0.338
Leptailurus serval	22.085	0.071	0.184	0.052
Lycaon pictus	21.950	0.071	0.421	0.338
Mellivora capensis	44.450	0.143	0.193	0.085
Panthera leo	15.085	0.049	0.650	0.650
Panthera pardus	15.085	0.049	0.197	0.080
Parahyaena brunnea	21.708	0.070	0.238	0.174
Proteles cristatus	25.458	0.082	0.281	0.236

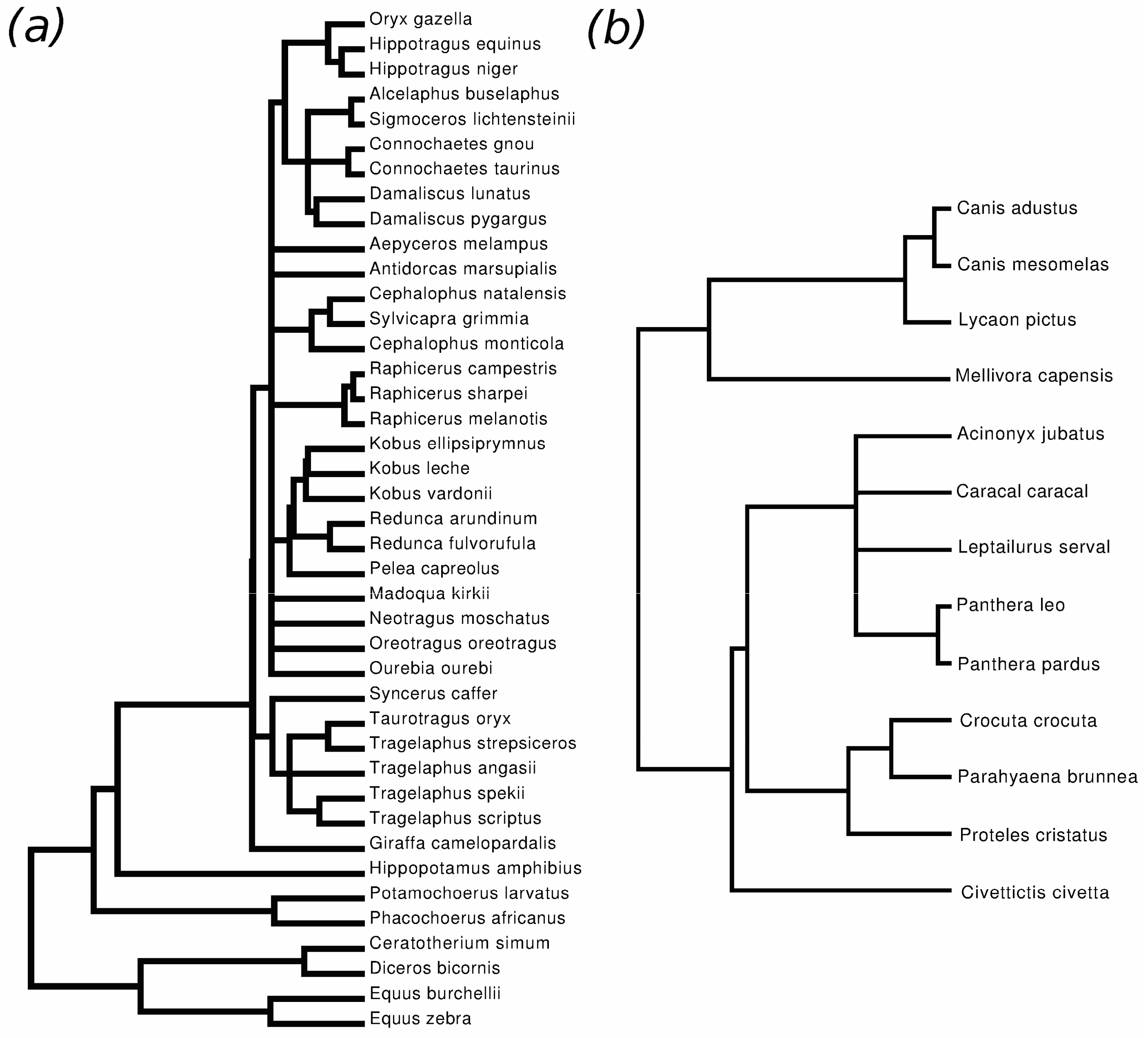

Fig. S1. Phylogenetic relationships among species of the southern African ungulate (a) and large carnivore (b) assemblages. Branch lengths are scaled to reflect the relative time since divergence. Phylogenetic relationships are derived from a complete mammal phylogeny (Bininda-Emonds et al. 2007) cropped to only contain species within the southern African ungulate and large carnivore assemblages.

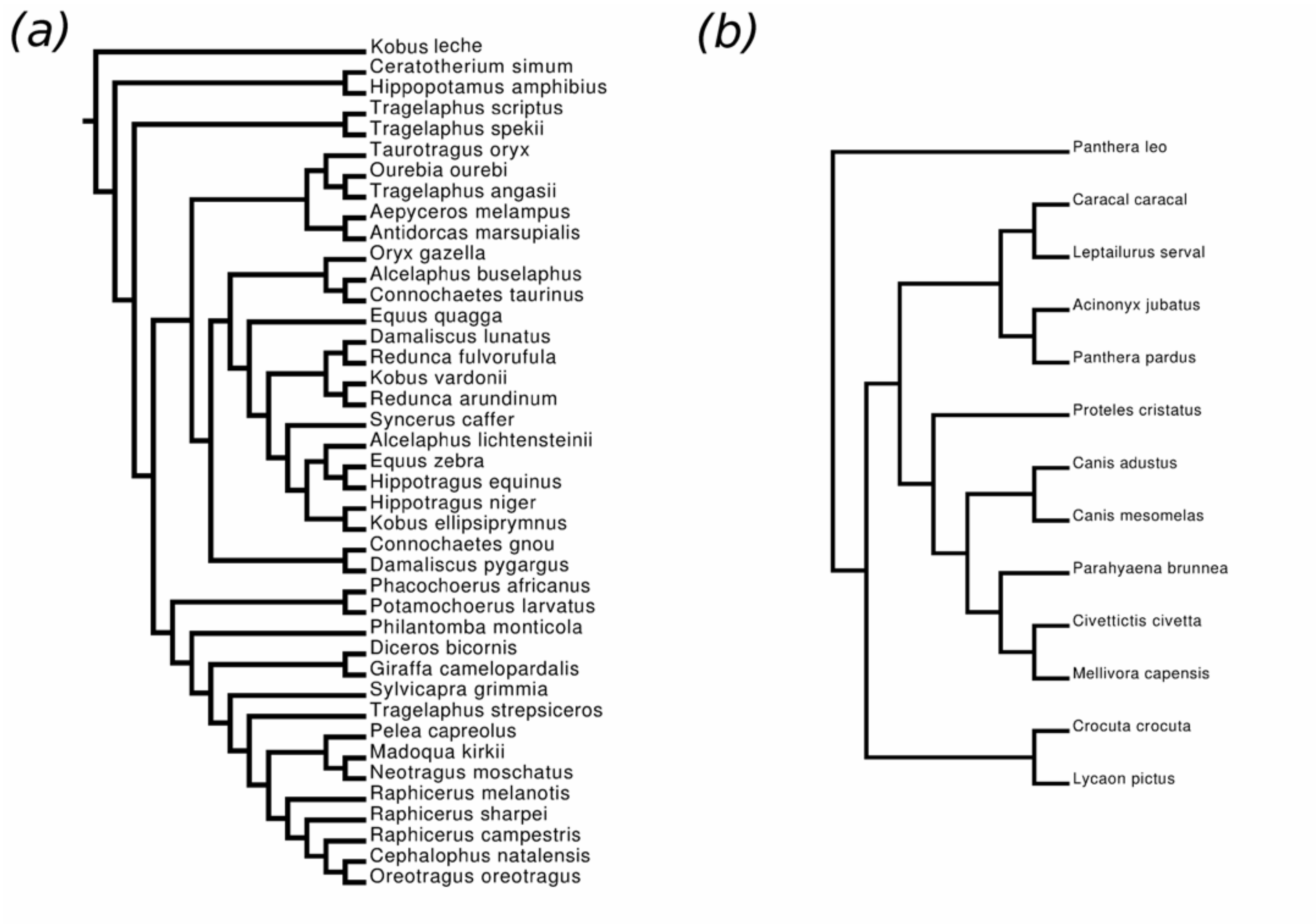

Fig. S2. Dendrograms visualizing functional relationships among species of the southern African ungulate (a) and large carnivore (b) assemblages. Branch lengths are scaled to reflect the relative time since divergence. Functional relationships were derived from trait matrices of traits relevant to herbivore (ungulates, Table S4) and predation (carnivores, Table S6) that were converted to Euclidean distance matrices that were clustered using the UPGMA (Unweighted Pair-Group Method using Arithmetic averages) clustering method.

References

Cain J. W., P. R. Krausman, and H. L. Germaine, 2004. *Antidorcas marsupialis*. *Mammalian Species* 753: 1-7.

Dalerum F. 2013. Phylogenetic and functional diversity in large carnivore assemblages. *Proceedings of the Royal Society of London, Series B Biological Sciences* 280: 20130049.

Jones, K. E., J. Bielby, M. Cardillo, S. A. Fritz, J. O'Dell, C. D. L. Orme, K. Safi, W. Sechrest, et al. 2009. PanTHERIA: a species-level database of life history, ecology, and geography of extant and recently extinct mammals. Ecology 90: 2648, Ecological Archives E090-184-D1.

Leuthold, B. 1979. Social organization and behaviour of giraffe in Tsavo East National Park. African Journal of Ecology 17: 19–34.

Richards M., and S. Shurter, 2006. Lowland nyala. Online fact sheet available from the The American Zoo and Aquarium Association's Antelope Taxon Advisory Group (TAG): http://antelopetag.com [accessed 2015-03-16]

Skinner, J. D. and R. H. N. Smithers. 1990. The mammals of the southern African subregion. 2nd ed. Pretoria: University of Pretoria.

Werdelin L. 1996. Carnivoran ecomorphology: a phylogenetic perspective. In *Carnivore behavior, ecology and evolution*, vol. 2, ed. J. L. Gittleman), 582–624. Ithaca: Cornell University Press.
